# Effect of Deposition Working Power on Physical Properties of RF-Sputtered CdTe Thin Films for Photovoltaic Applications

**DOI:** 10.3390/nano14060535

**Published:** 2024-03-18

**Authors:** Ana-Maria Răduță, Ana-Maria Panaitescu, Marina Manica, Sorina Iftimie, Vlad-Andrei Antohe, Ovidiu Toma, Adrian Radu, Lucian Ion, Mirela Petruta Suchea, Ștefan Antohe

**Affiliations:** 1 R&D Center for Materials and Electronic & Optoelectronic Devices (MDEO), Faculty of Physics, University of Bucharest, Atomiștilor Street 405, 077125 Măgurele, Ilfov, Romania; ana.raduta@fizica.unibuc.ro (A.-M.R.); anamaria.pam11@yahoo.ro (A.-M.P.); marina.manica@imt.ro (M.M.); sorina.iftimie@fizica.unibuc.ro (S.I.); vlad.antohe@fizica.unibuc.ro (V.-A.A.); thtoma72@yahoo.com (O.T.); adrian.radu@fizica.unibuc.ro (A.R.); lucian@solid.fizica.unibuc.ro (L.I.); 2 National Institute for Research and Development in Microtechnologies IMT-Bucharest, 023573 Voluntari, Ilfov, Romania; 3 Institute of Condensed Matter and Nanosciences (IMCN), Université catholique de Louvain (UCLouvain), Place Croix du Sud 1, B-1348 Louvain-la-Neuve, Belgium; 4 Center of Materials Technology and Photonics, School of Engineering, Hellenic Mediterranean University, 71410 Heraklion, Greece; 5 Academy of Romanian Scientists (AOSR), Ilfov Street 3, 050045 Bucharest, Romania

**Keywords:** cadmium telluride (CdTe) thin films, RF–magnetron sputtering (RF–MS), physical properties, current–voltage measurements

## Abstract

The main objective of this study was to determine the variation in the properties of cadmium telluride (CdTe) thin films deposited on a p-type Si substrate by the radio frequency magnetron sputtering technique at four different working powers (70 W, 80 W, 90 W, and 100 W). The substrate temperature, working pressure, and deposition time during the deposition process were kept constant at 220 °C, 0.46 Pa, and 30 min, respectively. To study the structural, morphological, and optical properties of the CdTe films grown under the mentioned experimental conditions, X-ray diffraction (XRD), scanning electron microscopy (SEM), atomic force microscopy (AFM), and optical spectroscopy were used. For a better analysis of the films’ structural and optical properties, a group of films were deposited onto optical glass substrates under similar deposition conditions. The electrical characterisation of Ag/CdTe/Al “sandwich” structures was also performed using current–voltage characteristics in the dark at different temperatures. The electrical measurements allowed the identification of charge transport mechanisms through the structure. New relevant information released by the present study points towards 90 W RF power as the optimum for obtaining a high crystallinity of ~1 μm nanostructured thin films deposited onto p-Si and optical glass substrates with optical and electrical properties that are suitable for use as absorber layers. The obtained high-quality CdTe nanostructured thin films are perfectly suitable for use as absorbers in CdTe thin-film photovoltaic cells.

## 1. Introduction

The best way to solve the problems of energy shortage and providing cleaner energy sources is given by photovoltaic (PV) technology. Among the A^II^-B^VI^ binary semiconducting compounds, CdTe is one of the most promising PV materials that can be used especially for applications in the field of thin-film solar cells, recording impressive conversion efficiencies of >22% [1,2,3].

There is a growing interest in the research community and industry in reaching efficiency limits approaching 28%, similar to GaAs solar cells, which have a similar bandgap and more or less the same cost [4,5]. Several material factors currently limit device efficiencies such as carrier recombination in bulk [6], grain boundaries, and interfaces. Several studies show that reducing carrier recombination with the contact layers at the CdTe interface may allow an increase in the device’s performance exceeding 25% efficiency [7]. To achieve 25% efficiency and >1 V for open-circuit voltage (V_oc_), research and development are needed to increase the minority carrier lifetime beyond 100 ns, to reduce the grain boundary and interface recombination, and to tailor band diagrams at the front and back interfaces [8]. Back contact optimisation also represents a potential solution to improve device’s efficiencies [9,10,11].

This material is highly attractive due to its following properties: CdTe is the second lowest-cost material after Si in the world of photovoltaic market and it has high absorption coefficient values (10^4^–10^5^ cm^−1^) with a direct band gap of 1.45 eV at room temperature (300 K), close to the ideal band gap for absorbing the maximum amount of the solar spectrum using one band gap p-n junction [12,13,14,15].

Various deposition techniques were used to prepare CdTe thin films, such as physical vapour deposition (PVD) [16], sputtering [17], vacuum evaporation [14,18,19,20,21,22], spray pyrolysis [23], pulsed laser deposition (PLD) [24,25,26,27], electrodeposition [28], molecular beam epitaxy (MBE) [29], close spaced sublimation (CSS) [30,31], etc. Many researchers have focused on using deposition techniques that are simple and less costly, and that can provide stability and good efficiency [32]. Among the fabrication methods, PVD techniques appear to be more popular due to its high deposition rate, low cost of operation, and low material consumption. It has been observed that TVE (thermal vacuum evaporation) is a suitable technique to be used for the deposition of CdTe nanostructured thin films; one remarkable example would be the results reported by O. Toma et al. in their study on the optical, morphological, and electrical properties of CdTe nanostructured thin films deposited by thermal vacuum evaporation [14]. The foremost deposition methods that are currently used commercially on a large scale are vapor-phase transport deposition, CSS, and sputtering [8]. CdTe thin-film solar cell absorber layer deposition generally requires raw material with 99.999% (5 N) CdTe purity. The CdTe films grown by different deposition processes can be a p- or n-type with a small degree of Cd or Te deficiency. The CdTe films deposited by magnetron sputtering usually have better grain distribution uniformity and a denser Cd-rich surface. Compared with other deposition methods, the deposition temperature in magnetron sputtering is lower, and it leads to a smaller grain size and higher surface densities. Overall, anion doping is more powerful in a cation-rich setting. In contrast, an anion-rich environment is more favorable for cation doping. It was also observed that the carrier lifetime of CdTe grown in a Cd-rich environment is usually higher than that in a Te-rich environment [33]. Inevitably, many defects are introduced during the CdTe deposition process, such as intrinsic, impurity and electronic defects. Intrinsic defects cause not only crystalline lattice distortion but also changes in the electrical properties of the material. Understanding the fundamental properties of the materials and the optimisation of growth conditions are essential for obtaining devices with good performance.

The aim of the present work is to improve the quality of CdTe thin films by using RF–magnetron sputtering (RF–MS) as a reliable deposition method, in which one has the ability to control the deposition parameters more easily. This study’s purpose was to investigate the influence of RF power on the physical properties of thin CdTe films deposited on two types of substrates: optical glass and p-type Si. For this study, four different powers of 70 W, 80 W, 90 W, and 100 W have been selected.

The results from investigations employing techniques like GIXRD (grazing incidence X-ray diffraction), SEM (scanning electron microscopy), AFM (atomic force microscopy), UV–VIS (ultraviolet–visible) spectroscopy, and I–V characteristics highlight the significant potential of these nanostructured thin films to serve as absorbent layers in ultra-thin-film solar cells. This work’s results add important experimental information to existing studies regarding the optimisation of the properties of CdTe material in the RF magnetron sputtering growth process that, up to now, proved to be one of the most suitable techniques for achieving CdTe thin-film layers that can be used as absorber layers in real-life, large-scale applications. Even though the literature on CdTe deposition is extensive, there are only a few studies discussing significant growth parameters and demonstrating reproducibility. Therefore, this study adds value to the existing international database on this specific subject. New relevant information released by the present study points towards 90 W RF plasma power as an optimum for obtaining a high crystallinity of ~1um thin films deposited onto p-Si and optical glass substrates with optical and electrical properties that make them suitable for use as absorber layers.

## 2. Materials and Methods

In this paper, CdTe nanostructured thin films were deposited by the RF–magnetron sputtering technique at an RF generator frequency fixed at 13.56 MHz [34,35,36,37]. The magnetron sputtering equipment was purchased from Tectra GmbH (Frankfurt, Germany), accommodating one sputtering gun and with capabilities of both radio-frequency (RF) and direct-current (DC) deposition modes. The CdTe targets were purchased from FHR Anlagenbau GmbH (Ottendorf-Okrilla, Germany) with their geometrical features being 50.8 mm in diameter and 3 mm in thickness. The purity of CdTe targets was 99.99%. The CdTe nanostructured thin films were sputtered on p-type Si (doped with Br) substrates purchased from Siegert Wafer GmbH (Aachen, Germany). The p-Si substrates used have a resistivity of 5 mΩ/sq. and (111) crystalline orientation. The second type of substrate used for deposition of CdTe films was optical glass substrates purchased from Merck KGaA (Darmstadt, Germany). Substrates were heated at a temperature of 220 °C during depositions. The substrates were ultrasonically cleaned in acetone, isopropyl alcohol, and deionised water for 15 min and dried over nitrogen flow. As sputtering gas, pure Ar was used and the chamber pressure was maintained at 0.46 Pa during the deposition process. To remove eventual impurities, the CdTe target was pre-sputtered for 10–15 min. The most important parameters used for the deposition of CdTe nanostructured thin films were as follows: a target-to-substrate distance of 9 cm, RF power ranging from 70 W to 100 W, and a deposition time of 30 min. Thus, films with different thicknesses were obtained. Subsequently, the RF-sputtered CdTe nanostructured thin films were subjected to a thermal treatment under nitrogen gas flow using an oven from Nabertherm GmbH (Lilienthal, Germany). The prepared films were thermally treated at 450 °C with a 5 °C/min heating rate, and were kept on a bearing level for 30 min.

The structural properties of the fabricated samples were investigated by GIXRD with a diffractometer from Bruker (Ettlingen, Germany), model D8 Discover (using CuKα radiation at λ = 1.54 Å). All the GIXRD measurements were performed at an angle of 2°. The scattered intensity was scanned in the 2θ range between 20° and 60° with a step size of 0.02°/s at room temperature. The topography of the surface of fabricated CdTe samples was analysed by AFM, in non-contact mode, using an AFM microscope from A.P.E. Research (Trieste, Italy), model A 110-SGS. At the same time, based on the results obtained from AFM, the distribution of the peaks over the investigated surfaces was analysed using computed values for root mean square, skewness, and kurtosis parameters using the Gwyddion software package (version 2.52, Brno, Czechia). Morphological investigations of CdTe nanostructured thin films were performed by scanning electron microscopy (SEM) using a Tescan Vega XMU-II (Brno, Czechia) instrument, operating at an accelerating voltage of 30 kV and a working distance (WD) of about 7 mm in high vacuum. From the cross-section SEM images, the thicknesses and surface uniformities of all the samples were estimated.

The optical investigations were made using a spectrophotometer from PerkinElmer (Waltham, MA, USA), model Lambda 750 UV/VIS/NIR. The optical transmission and optical absorption of the CdTe films were determined in the wavelength region between 400 nm and 1800 nm, in air and at room temperature. Thicknesses of CdTe films were computed from optical transmission data. Additionally, current–voltage characteristics of the Ag/CdTe/Al structure at different temperatures were used to investigate the electrical properties. These were recorded using a computer-controlled experimental setup from Keithley (Beaverton, OR, USA).

By investigating the structural, morphological, and optical properties, it was observed that the CdTe sample deposited at 90 W is the most suitable for use as an absorber layer. Thus, the sample obtained through sputtering at 90 W onto an optical glass substrate was coated with a thin layer of silver (Ag) that was thermally evaporated in vacuum using an evaporation equipment, model AV 500 FAN (Bucharest, Romania), with the Ag layer serving as the back electrode. For the top contact, the aluminium (Al) fabricated via VTE was used to close the “sandwich” structure Ag/CdTe/Al. The latter was employed for electrical characterisation of the CdTe semiconducting thin film. Both electrical contacts were evaporated at 0.1 × 10^−3^ mbar working pressure for 1 min and, afterwards, the structure was subjected to thermal treatment for 10 min at 100 °C.

**Figure 1:**
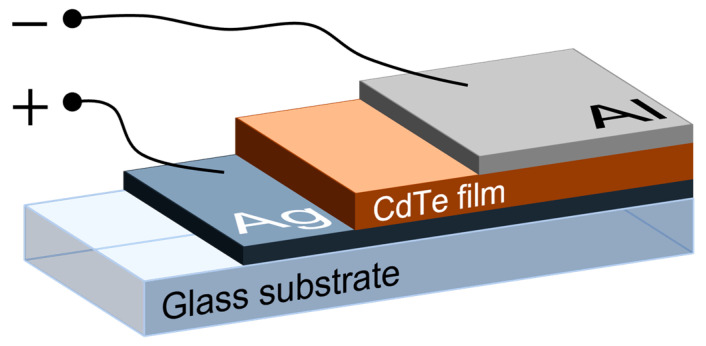
Schematic representation of the Ag/CdTe/Al “sandwich” structure.

The experimental set-up consisted of a source meter from Keithley (Beaverton, OR, USA), model DM-2400, and a homemade heater for the sample holder [38]. The temperatures of the samples were measured using a thermocouple K-type chromel-alumel (with 25 °C/mV) placed on the sample. Figure 1 presents the schematic representation of the Ag/CdTe/Al structure.

## 3. Results

### 3.1. Structural Analysis by XRD

**Figure 2:**
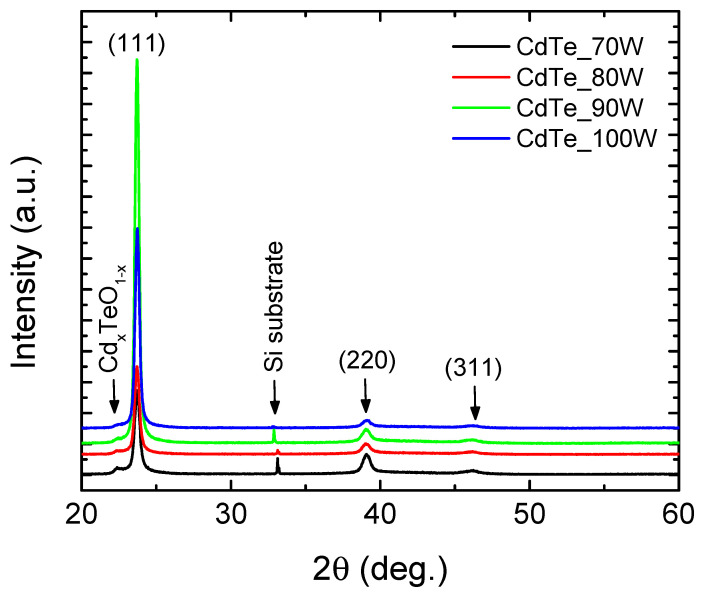
GIXRD patterns of CdTe nanostructured thin films deposited at different RF powers.

As mentioned above, the structural properties of CdTe nanostructured thin films were, at first, analysed by using grazing incidence X-ray diffraction. All of the GIXRD measurements were performed at an angle of 2°. The scattered intensity was scanned in the 2θ range between 20° and 60° with a step size of 0.02°/s at room temperature. Figure 2 shows the diffraction patterns recorded in the GIXRD configuration obtained from the films deposited on the p-Si substrate at different RF powers of 70 W, 80 W, 90 W, and 100 W. According to Figure 2, the films are polycrystalline and present good crystallinity. They are preferentially oriented with (111) crystallographic planes parallel to the surface (the noticeable peak being at 2θ = 23.7°). Also, the other peaks of cubic CdTe at 2θ = 39.06° and 2θ = 46.16° were observed, corresponding to the reflections from the (220) and (311) planes, respectively.

The predominant growth direction of CdTe nanocrystallites on the (111) plane in GIXRD spectra may be attributed to the comparatively lower surface energy on this plane compared to the (220) and (311) planes. This discrepancy could stem from an augmented diffusivity of atoms in the substrate [38]. As the RF power increases, there is an observable rise in the intensity of the (111) peak, indicating an enhancement in crystallinity within the CdTe films. Concurrently, the peaks associated with the (220) and (311) crystalline planes demonstrate a decrease.

The intensity of the diffraction peak corresponding to the (111) orientation increases significantly as the RF power increases to 90 W, where this behaviour relates to better crystallinity of the sample, decreasing at 100 W. This effect may be attributed to an improved electron mobility promoted by the increase in the kinetic energy of the adatoms sputtered on the surface up to an optimum value, which is required for obtaining films with a highly crystalline structure.

The appearance of a small peak of Cd_x_TeO_1-x_ at 2θ = 22.36° was also noted and the presence on the surface or in the material of these oxygen-containing compounds was not expected, but it is due either to the fact that during the deposition process there were oxygen atoms, or to the fact that the target is not made entirely of CdTe, containing a small amount of oxygen.

**Figure 3:**
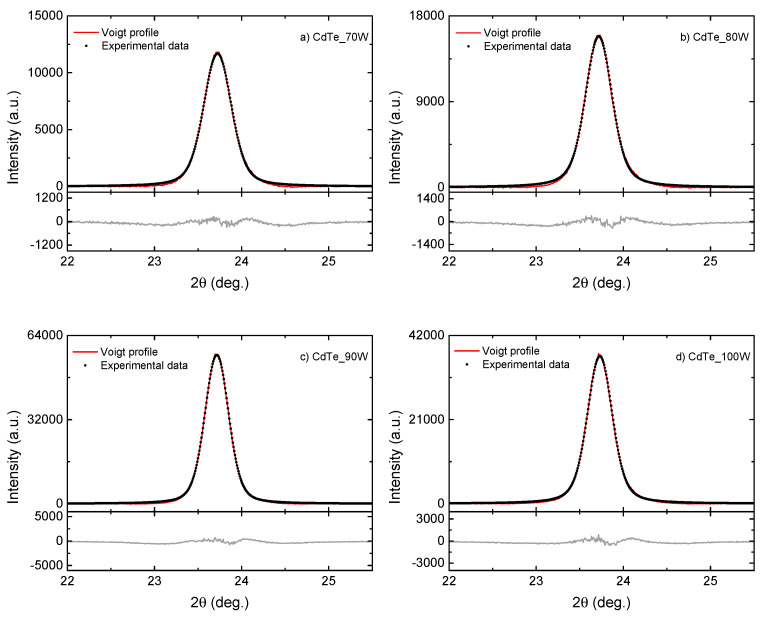
Deconvolution using Voigt profiles of the (111) diffraction peak corresponding to (**a**) CdTe_70W; (**b**) CdTe_80W; (**c**) CdTe_90W; and (**d**) CdTe_100W deposited by RF–magnetron sputtering on a p-Si substrate. The peak XRD profiles were recorded in Bragg–Brentano theta–theta geometry. The experimental data have been processed with the Voigt function, and the associated residual is shown at the bottom of each graph.

It is already known that, due to the supplementary broadening of diffraction peaks, GIXRD experiments are not adapted to obtain quantitative information, and, for this reason, the microcrystalline properties of CdTe nanostructured thin films were investigated by performing a profile analysis on the (111) reflection in Bragg–Brentano theta–theta geometry. The experimental results and line profiles as obtained by analytical fitting using Voigt profiles are shown in Figure 3. The parameters associated with the crystallinity quality of CdTe nanostructured thin films, such as the crystalline coherence length (reflecting the crystallite size) $D_{ef}$ and mean-square strain ${<\varepsilon^{2}>}^{1/2}$ induced by mechanical stresses developed at the microscopic level during film growth, have been determined using the integral breadths $\beta_{G}$ and $\beta_{L}$ of the Gauss and Lorentz components of the Voigt profiles, after correction for instrumental broadening [35,39]. The crystallite size $D_{ef}$ and mean-square strain ${<\varepsilon^{2}>}^{1/2}$ were calculated, considering that the first is given by the Lorentzian integral breadth (see Debye and Scherrer’s formula), while the micro-strain distribution is Gaussian, as follows:

(1) $$D_{ef}=\frac{0.9\lambda }{\beta_{L}cos\theta }$$

(2) $$\beta_{G}={2(2\pi )}^{1/2}{<\varepsilon^{2}>}^{1/2}\;tan\theta$$

The crystalline structural parameters that were determined experimentally for CdTe nanostructured thin films are displayed in Table 1. It was expected that the average crystallite size would increase when the RF-sputtering power increased for CdTe nanostructured thin films deposited on a p-type Si substrate, but in the case of the present samples, a random variation was observed [40].

The values of the lattice parameter (a) for the cubic phase range from around 6.489 to 6.493 Å are also given in Table 1. All these films show relatively low values of the lattice strain, which suggests that the films may be subjected to tensile stress in the plane parallel to the substrate surface. These results are a consequence of internal stress on the crystallites and might be also due to the lattice mismatch and differences in the thermal expansion coefficients of glass, which are as follows: ~6–7 × 10^−6^/K, Si ~2.6–3.3 × 10^−6^/K, and CdTe ~5–6 × 10^−6^/K, respectively [41,42,43]. As these nanostructured films are polycrystalline with a thickness of hundreds of nm, the structural configurations causing intrinsic residual stresses may also include the contribution of the microstructure, including imperfections between the boundaries of grains and grain columns, voids between grains, and other similar micro-scale defects in the thin film together with the atomic-level point defects, misfits, dislocations, impurity incorporation, etc. All these structural issues are the result of non-equilibrium growth conditions and/or the incorporation of impurities into the growing thin-film layer. Atomic-level defects in the lattice structure and imperfections in the microstructure can both cause elastic deformations of the thin-film material layer, thereby resulting in intrinsic residual stress, and a trial to discriminate between each contribution would be inaccurate. 

It can be also noticed from Table 1 that the highest mean crystallite size and lowest lattice strain values correspond to the CdTe films sputtered at 90 W RF plasma power. 

### 3.2. Morphological Investigation by SEM

**Figure 4:**
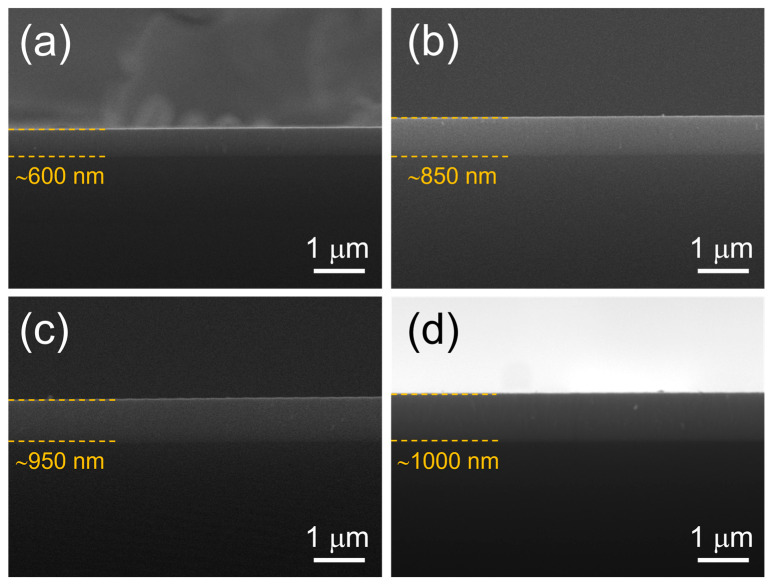
Cross-section scanning electron microscope (SEM) micrographs of the CdTe nanostructured thin films sputtered on p-Si substrate for 30 min, at the following working RF powers: (**a**) 70 W; (**b**) 80 W; (**c**) 90 W; and (**d**) 100 W. Corresponding estimated thickness SEM measurements for the CdTe nanostructured thin films are indicated.

Morphological investigations of the CdTe nanostructured thin films deposited by RF–magnetron sputtering on p-Si substrates were also carried out in terms of SEM cross-sectional analysis in secondary electron (SE) mode imaging. Figure 4 highlights that all sputtered CdTe nanostructured thin films at different working RF powers are compact and suggests quite conformal growth on the p-Si substrate.

By choosing an optimal distance between the target and substrate during deposition, the surfaces do not show damage and there are no drops formed during the sputtering process. It is noteworthy that the samples were carefully prepared to minimise the charging effects by dicing the CdTe-sputtered p-Si substrates and covering their section with conducting carbon tape. The mean values obtained for each measured thickness are between 583 nm and 983 nm, being close to the values estimated from optical transmission spectroscopy data. The mean values of thickness obtained from the cross-section measurements are given in Table 2.

**Figure 5:**
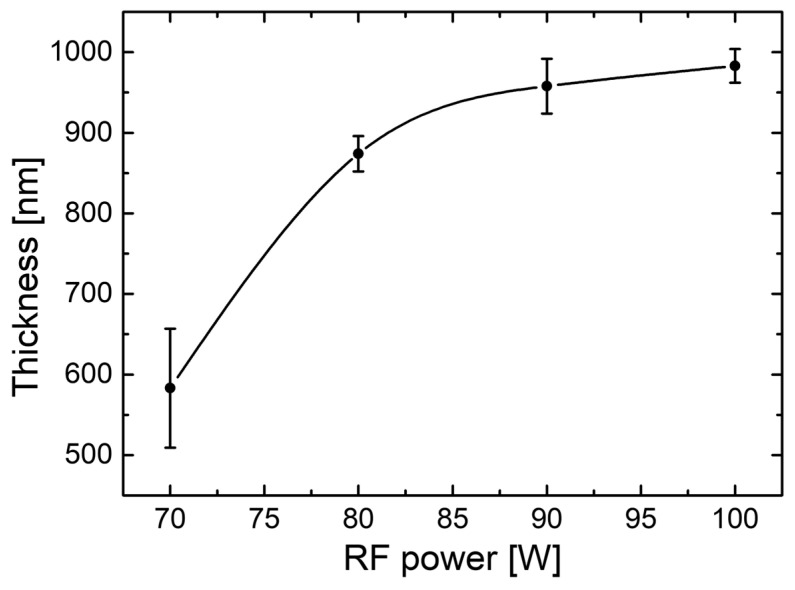
Variation in CdTe nanostructured thin films’ thickness as a function of RF plasma power, under the manufacturing sputtering conditions specified in the text.

According to sputtering theory, the sputtering deposition rates of films are proportional to the product of the sputtering rate of the target and the density of the Ar ions. Therefore, the sputtering rate of the target also increases. Consequently, the growth rate of the film and, hence, the thickness of the film, are expected to increase with the increase in the RF power [32]. The experimental thickness data from the cross-section SEM imaging analysis are represented in Figure 5. They show the dependence of the CdTe nanostructured thin films’ thickness on the RF power during deposition. One can observe that the CdTe nanostructured thin films’ thickness values tend to increase with the RF power as expected, and this is primarily due to the increase in kinetic energy and velocity of the particles (by scattering away from the target) leading to a higher deposition rate. A slight saturation is observed at 90 W and 100 W and this may be associated with the excessively high energy of the ions injected into the target, which leads to an energy and quantity loss for the Ar ions. As has been shown in other studies, this behaviour leads to a decrease in the deposition rate [44].

### 3.3. Surface Topography Analysis by AFM

**Figure 6:**
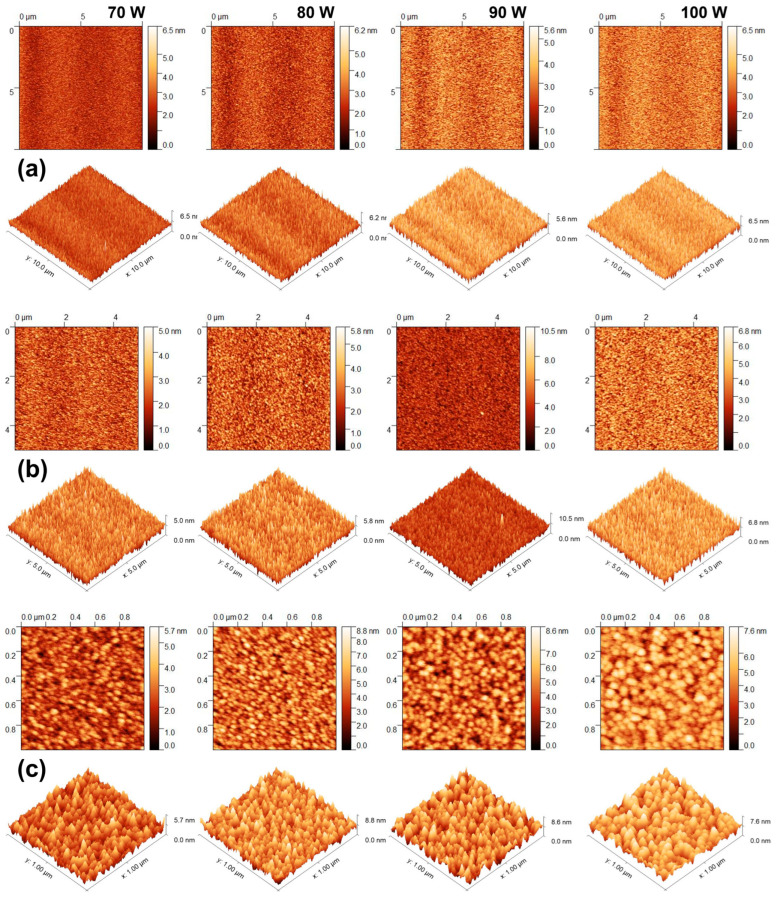
The 2D and corresponding 3D atomic force microscope (AFM) images of the CdTe nanostructured thin films sputtered at various RF powers for the scanned areas of 10 × 10 μm^2^ (**a**), 5 × 5 μm^2^ (**b**), and 1 × 1 μm^2^ (**c**).

Cross-section SEM morphological investigations can be combined with the AFM technique in order to achieve a complete view over the CdTe nanostructured thin films’ growth quality. The surface topographies of the CdTe nanostructured thin films deposited at different RF powers were analysed by AFM in non-contact mode. Figure 6 shows the 2D and 3D AFM images of the surface topography of the CdTe nanostructured thin films sputtered at different RF powers. The AFM scanned areas for each sample were as follows: 1 µm^2^ (upper row); 5 µm^2^ (middle row); and 10 µm^2^ (lower row), acquired in order to provide insight into the films’ surface quality, at lower and larger scales, respectively. As one can observe from the AFM images, the polycrystalline nature of the CdTe nanostructured thin films is confirmed by the granular structure of the surface, consisting of grains with sizes in the order of tens of nanometres. The average size of the surface grains is obviously in agreement with the crystallites’ size variation observed from the GIXRD data analysis. The grains present on the CdTe film obtained from the deposition at 80 W RF power show the smallest size and the highest z-range, although the surface RMS seems to increase with an increasing RF sputtering power. Also, it can be observed that the film grown at 100 W RF power shows a smoother surface and a more uniform surface grain size distribution than the film obtained from the 90 W for power growth. This observation remains valid at a low scan size as well as at a high scan size for all CdTe nanostructured thin films (Figure 6).

**Figure 7:**
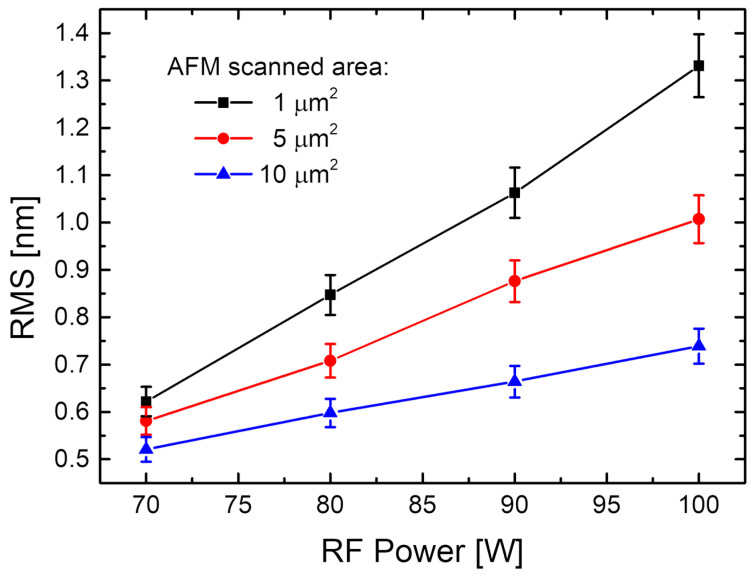
Root mean square (RMS) roughness as a function of the different RF powers for the sputtered CdTe nanostructured thin films, at the three scanned areas of 10 × 10 μm^2^ (blue triangles), 5 × 5 μm^2^ (red circles), and 1 × 1 μm^2^ (black squares).

For a better understanding of the surface morphology from AFM characterisation, the surface roughness morphology parameters, surface roughness, and statistical parameters such as the skewness parameter (Ssk)—which is a third-order statistical parameter—were used to describe how symmetric a statistical distribution is (one can have an almost perfect Gaussian distribution if the statistical distributions have Ssk values close to zero) and the kurtosis parameter (Sku)—a fourth-order statistical parameter used to describe how sharp or how broad a statistical distribution is (one can have broader distributions if the values are below 3 or sharp distributions in the case of values above 3) [34]—was estimated. All these parameters were evaluated using specialised open-source software for scanning probe microscopy data processing (Gwyddion). As can be seen from Table 3, the root-mean square (RMS) roughness values for all the RF-sputtered CdTe nanostructured thin films deposited on p-Si substrates at different working powers are relatively low and increase when the RF power increases. This behaviour was also observed in a plot of RMS roughness as a function of the RF power (Figure 7).

A negative Ssk value signifies that the surface predominantly comprises valleys, whereas a positive skewness indicates that it consists mainly of peaks and asperities. Sku measures the sharpness of profile peaks, with a negative Ssk indicating deeper voids on the surface. If the excess Sku value is negative, the roughness distribution is termed platykurtic, characterised by a thinner tail than a normal distribution. Elevated Sku values suggest a distribution that is more peaked than normal.

As one can observe from the surface parameter estimations presented in Table 3, all CdTe nanostructured thin films obtained using various RF sputtering powers show low values of surface RMS, even at the smallest scan size, that remain below 1.4 nm. The RMS variation with the different RF powers for the sputtered CdTe nanostructured thin films, at the three scanned areas of 10 × 10 μm^2^ (blue triangles), 5 × 5 μm^2^ (red circles), and 1 × 1 μm^2^ (black squares), is represented in Figure 7. It is obvious that the RMS increases with the increase in the RF power. Exploring surface parameters at the various scan sizes while looking at the 2D and 3D AFM images presented in Figure 6, it can be observed that, as the scan size decreases, all the films conserve their homogeneity and the dimensional distribution of the grain sizes as well as the topology of the peaks/valleys are quite uniform. As can be seen from the AFM images, the best peaks/valleys distribution corresponds to the film grown at 90 W RF power. This observation is consistent with the XRD observations. The influence of the thickness uniformity and surface morphology of the sputtered CdTe nanostructured thin films plays an important role in their electrical and optical properties and, implicitly, on the PV device efficiency. A higher sputtering power is more likely to promote increased kinetic energy of the CdTe atoms and further improvement of the mobility and diffusion of the CdTe atoms on the substrates, leading to bigger particles with uniform distribution on the substrate. However, a further increase in the sputtering power to 100 W seems to lead to a more inhomogeneous grain distribution that may be attributed to excessive sputtering power that affects the growth of the CdTe polycrystalline films on the substrate. These observations are consistent with previous observations in studies of CdTe growth at much lower RF sputtering powers [45]. It is worth mentioning that the RMS values obtained for the present films are much smaller than any other values reported in the previously mentioned studies.

### 3.4. Optical Characterisation by UV–VIS Spectroscopy

**Figure 8:**
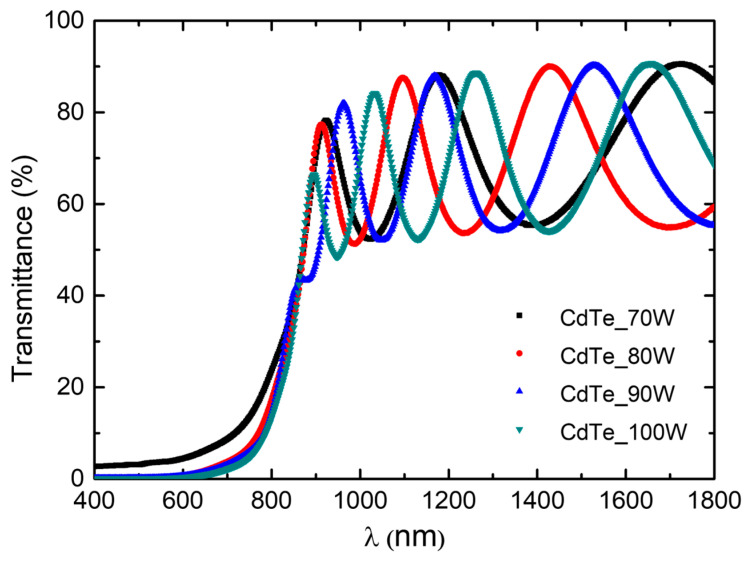
Optical transmission spectra of CdTe nanostructured thin films deposited on optical glass substrates.

The optical properties of CdTe nanostructured thin films deposited on optical glass substrates were investigated by optical transmission and absorption spectroscopy. All the spectra were recorded at room temperature. Optical transmission spectra were taken ranging from 400 to 1800 nm in wavelength and are presented in Figure 8. Transmission values higher than 65% can be observed in the 800–1800 nm region, confirming excellent transmittance properties to be employed as an absorber layer.

**Figure 9:**
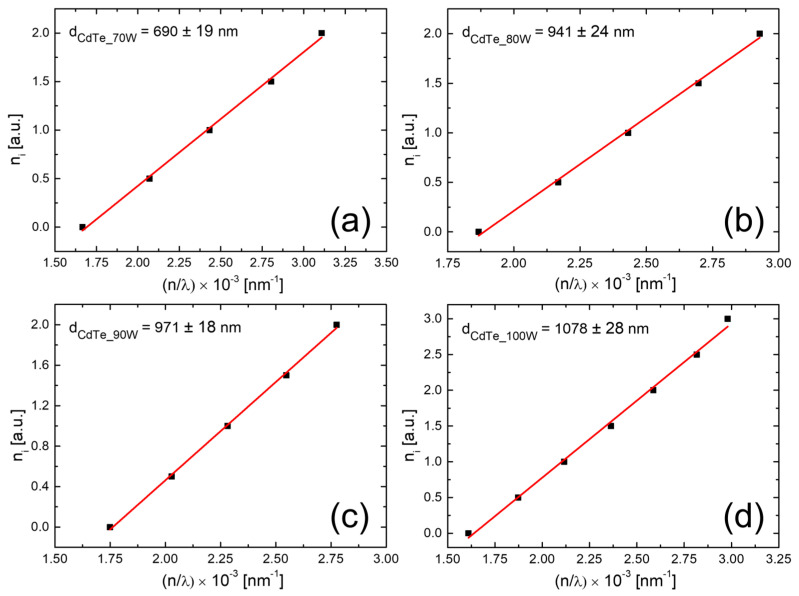
Determination of thicknesses corresponding to CdTe nanostructured thin films sputtered onto optical glass substrates at the RF plasma power of 70 W (**a**), 80 W (**b**), 90 W (**c**), and 100 W (**d**).

To determine the thicknesses of the samples, Bragg’s law was used, where *n_i_* is a function of *n*/*λ* where *n_i_* is related to the minimum and maximum conditions specific for the interference effects that appeared in the NIR (near infrared) region, *n* is the refractive index of the material (2.67 in the case of CdTe), and *λ* is the wavelength associated with *n_i_*. The corresponding values for the thicknesses of each CdTe thin film are represented in Figure 9, and a good agreement with the cross-SEM measurements of thickness is observed. From the absorption spectra, the optical band gap values corresponding to the CdTe nanostructured thin films’ material were calculated. To achieve this, Tauc plots were used, more precisely the dependence of the absorption coefficient on incident photon energies in the case of direct band gap semiconductors near the fundamental absorption edge, which is described by the subsequent equation:

(3) $$\alpha \left(h\nu \right)=\frac{A{\left(h\nu -E_{g}\right)}^{1/2}}{h\nu }$$

where *A* is a constant, *α* is the absorption coefficient, *h* is the Plank’s constant, *υ* is the frequency of radiation, and *E_g_* is the optical band gap corresponding to the *Γ* point of the first Brillouin zone. As one can see from Equation (3), *n* is an exponent that is dependent on the transition type (in the case of CdTe nanostructured thin films, *n* = 1/2 because it corresponds to a direct allowed transition).

**Figure 10:**
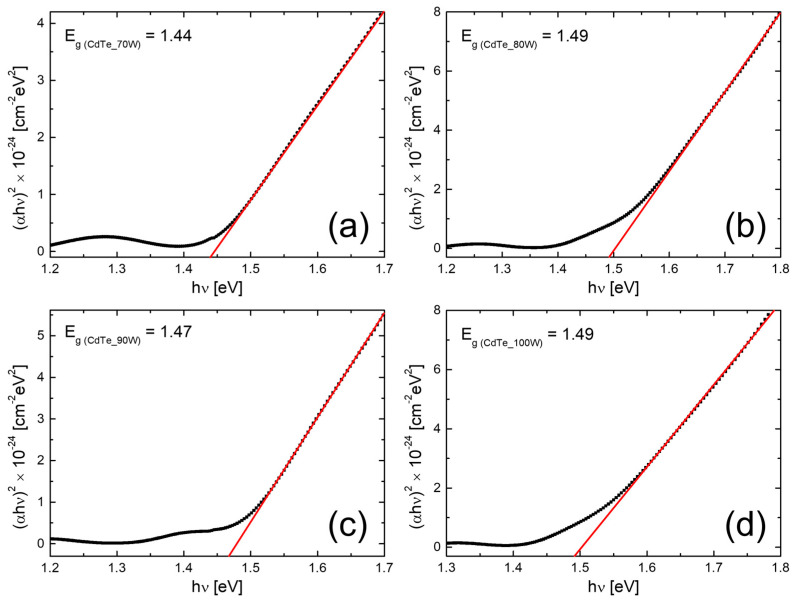
(**a**–**d**) Plots of (αhυ)^2^ vs. hυ for CdTe nanostructured thin films deposited on optical glass at different RF powers.

The bandgap calculation was carried out by extrapolating the straight-line portion of the *(αhυ)^2^* vs. *hυ* graph on the *hυ* axis at *α = 0*, and the plots are shown in Figure 10. The values of the *E_g_*, determined by fitting of the experimental data with Equation (1), are collected in Table 4.

It can be seen that the bandgap does not change significantly and remains in the range of 1.44–1.49 eV, being extremely close to the values that were previously reported in the literature. Moreover, as the film thickness increases, the optical band gap values of the CdTe nanostructured thin films vary, most likely in correlation with the crystallite size, as previously reported [14].

### 3.5. Electrical Characterisations

**Figure 11:**
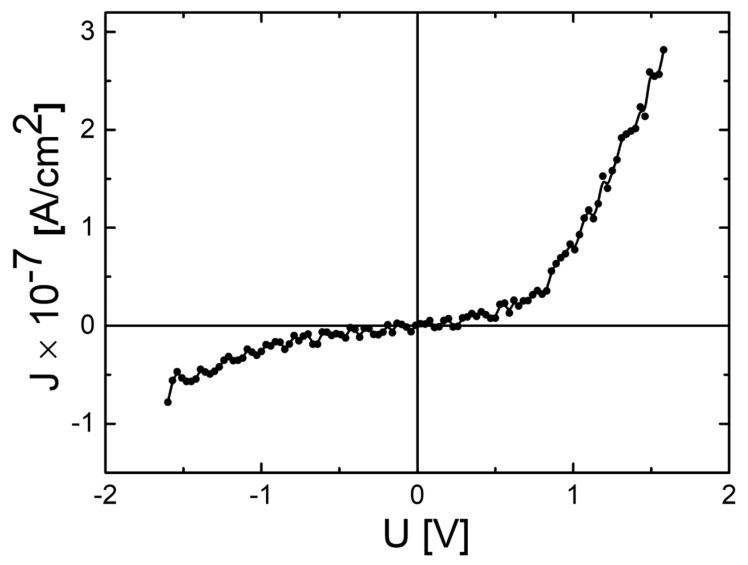
Current density–voltage (J-V) dark ambipolar characteristic recorded at a temperature of 316 K, for the Ag/CdTe/Al “sandwich” structure.

The Ag/CdTe/Al “sandwich” structure fabricated with CdTe thin film deposited by RF magnetron sputtering at a power of 90 W (deposition details are available in Section 2) was electrically contacted to study the transport mechanism of the charge carriers into the thin film of CdTe. The ambipolar current density–voltage (J-V) characteristic showing a non-linear behaviour with a relatively low asymmetry, is shown in Figure 11.

**Figure 12:**
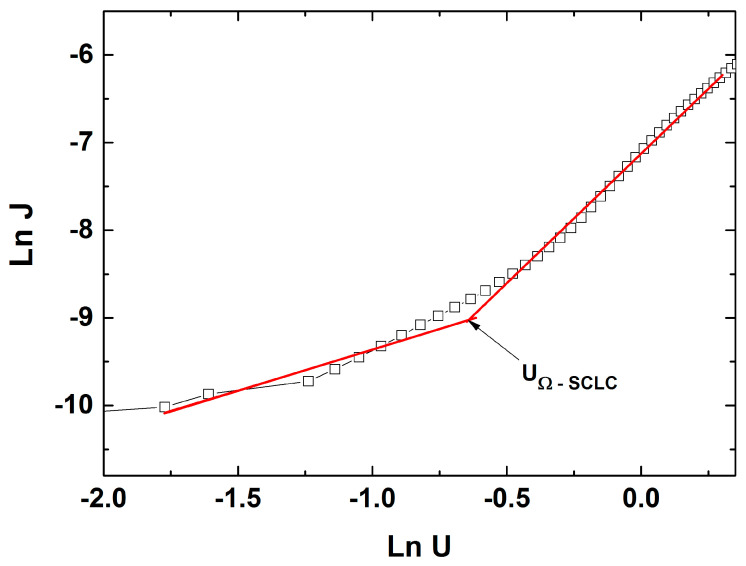
Logarithmic fit of ln J versus ln U for the Ag/CdTe/Al structure at forward bias, emphasizing the two linear regions corresponding to different conduction mechanisms through the structure, separated by the transition voltage.

To understand the charge transport mechanism through the Ag/CdTe/Al structure and to observe the presence of different conduction mechanisms along the same type of bias, in Figure 12, the forward bias (J-U) characteristics were plotted in a logarithmic scale. Choosing the most experimental points from a range of voltages for the linear regressing fit in such a way that the coefficient of regression would be higher than 0.9, two straight lines were obtained with different slopes. As one can see, two straight lines with different slopes emphasizing two regions were obtained, each related to a corresponding charge transport mechanism. For the first region, at low injection levels (low applied voltages), the slope of the linear plot is near unity (0.93); therefore, it shows a typical ohmic conduction mechanism, assured by the equilibrium charge carriers. For the region at high injection levels (high voltages), above the defined transition voltage (*U_Ω-SCLC_ = 0.53 V*), the slope is almost 3 (2.96), indicating a conduction mechanism based on space-charge-limited conductivity (SCLC) in the presence of an exponential trap distribution. The latter conduction mechanism is generally encountered in semiconducting materials with a low carrier mobility, thus having a high resistivity, usually A^II^–B^VI^ compounds such as CdS [46], ZnTe [37], and CdTe [14]. The fitting of experimental data was carried out following the relationships describing these two conduction mechanisms [24,47].

At low voltages, the charge transport mechanism is attributed to the equilibrium charge carriers; therefore, the current density–voltage characteristic is linear and obeys Ohm’s law:

(4) $$J_{ohmic}=p_{0}q\mu \frac{U}{d}=q\mu N_{V}\frac{U}{d}e^{-\frac{E_{F}-E_{V}}{k_{B}T}}$$

where *p*_0_ is the concentration of thermally activated free charge carriers at equilibrium, *q* is the elementary charge, *μ* is the mobility of holes, *U* is the applied voltage, *d* is the thickness of the CdTe film, *N_V_* represents the effective density of states in the valence band (VB), *E_F_ − E_V_* is the separation between the equilibrium Fermi level and the VB, *T* is the absolute temperature, and *k_B_* is Boltzmann’s constant. Above a specific voltage (*U_Ω-SCLC_ =* 0.53 V), there is a transition from the equation obeying an ohmic law to an equation corresponding to SCLC in the presence of exponential trap distribution. The equation that describes the presence of exponential traps present in the band gap of CdTe is as follows:

(5) $$\rho \left(E\right)=\frac{N_{t}}{k_{B}T_{C}}e^{-\frac{E}{k_{B}T_{C}}}$$

where *N_t_* is the number of effective trap levels per volume and *E* represents the traps’ depth level. The latter equation emphasises that the more the energy increases, the more the speed of the trap density per unit energy decreases. At high applied voltages, the current density follows the equation below.

(6) $$J_{SCLC-exp}=q\mu N_{V}\frac{\epsilon^{\gamma }}{{\left(qN_{t}\right)}^{\gamma }}\frac{U^{\gamma +1}}{d^{2\gamma +1}}$$

where *ε* is the dielectric constant of CdTe, and *γ = T_C_*/*T* is the ratio between the characteristic temperature and the ambient temperature. One can easily observe in Figure 12 that the ohmic conduction mechanism becomes dominant between 0.15 and 0.5 V and above the transition voltage (evaluated at 0.53 V), between 0.6 and 1.25 V, the transport through the Ag/CdTe/Al structure is based on the SCLC in the presence of the exponential trap distribution.

**Figure 13:**
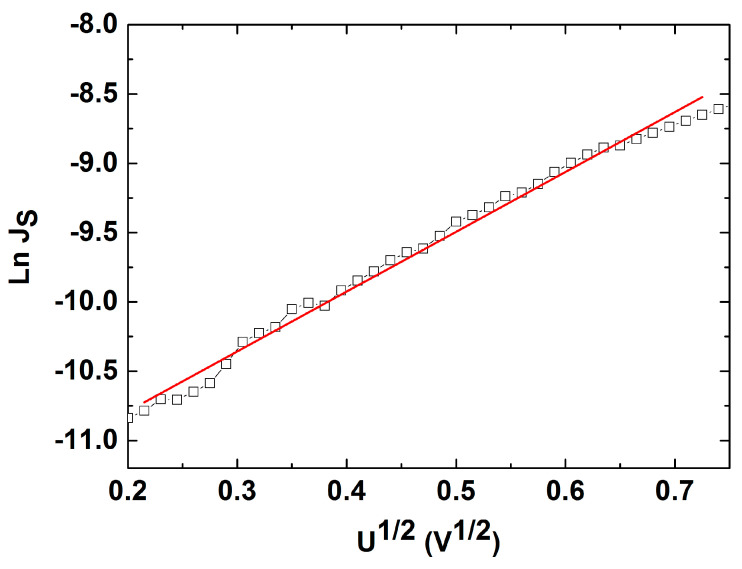
Logarithmic J_S_ as a function of the U^1/2^ characteristic at reverse bias that allows the determination of the depletion layer w.

**Figure 14:**
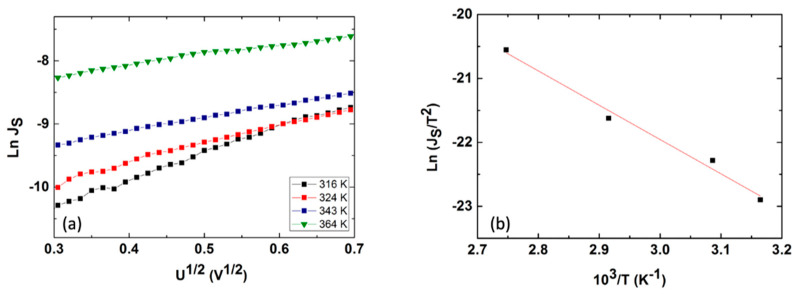
(**a**) Logarithmic J_S_ vs. U^1/2^ characteristics recorded at four temperatures, 316K, 324K, 343K, and 364K, that allowed the evaluation of the Schottky barrier at the Al/CdTe interface as Φ=0.47 eV by using the displayed (**b**) dependence of $\ln\left(\frac{J_{s0}}{T^{2}}\right)$ vs. $\left(\frac{10^{3}}{T}\right)$.

When applying a reverse bias (the third quadrant from Figure 11), the dominant transport mechanism through the Ag/CdTe/Al structure is based on the Schottky effect at a barrier potential. In this situation, the current density obeys the following law:

(7) $$J_{s}=A^{*}T^{2}e^{-\frac{\Phi }{k_{B}T}\;}e^{\frac{\beta_{S}\sqrt{U}}{k_{B}T\sqrt{w}}}=J_{s0}e^{\frac{\beta_{S}\sqrt{U}}{k_{B}T\sqrt{w}}}$$

where A^*^ is the Richardson constant for semiconductors ${(A}^{*}=\frac{4\pi qm^{*}K^{2}}{h^{3}})$, $\beta_{S}=\sqrt{\frac{q^{3}}{4\pi \varepsilon_{0}\varepsilon_{r}}\;}$ is the Schottky coefficient, *w* is the depletion region, and *Φ* represents the height of the Schottky barrier. Figure 13 illustrates the logarithmic dependence of the saturation current density on the voltage at reverse bias recorded at 316K and allows for the evaluation of the depletion layer *w*, i.e., obtained as 10 nm for this temperature. In order to provide a determination of the Schottky barrier, we have recorded four current density–voltage characteristics at temperatures of 316K, 324K, 343K, and 364K, as displayed in Figure 14a. Figure 14b emphasises the linear dependence of $\ln\left(\frac{J_{s0}}{T^{2}}\right)$ vs. $\left(\frac{10^{3}}{T}\right)$ for all four temperatures, thus allowing the determination of the Schottky barrier at the Al/CdTe interface, i.e., 0.47 eV, where *J_S0_* is the intercept of each characteristic from Figure 14a, namely the saturation current density value at 0 V. There is indeed a difference in the experimentally computed value of *Φ* (0.47 eV) at the Al/CdTe interface in comparison to the theoretical value:

$$\chi_{CdTe}+E_{g\;CdTe}-\Phi_{Al}≅3.7\;eV+1.4\;eV-4.3\;eV≅0.8\;eV.$$

The inherent presence of surface states at the Al/CdTe interface that usually leads to the increase in donor charge carriers’ concentration at the interface, which decreases the barrier at the metal/semiconductor interface, can explain the difference in the experimental value of the Schottky barrier evaluated at 0.47 eV in comparison to the theoretical value.

## 4. Discussions

The present study reports an investigation of the RF power impact upon the morphological, structural, and optical properties of sputtered CdTe nanostructured thin films. The CdTe nanostructured thin films were prepared via the RF–MS technique by varying the deposition power from 70 W up to 100 W. The structural investigation has revealed that all of the samples possess good crystallinity and a preferential orientation with (111) crystallographic planes parallel to the surface. In addition, the typical structural parameters (crystallite size, mean-square strain, and experimental value of the lattice constant) were determined for each fabricated sample. A tendency was observed for increases in the crystallite size that were correlated with the increasing of the RF sputtering power, in agreement with the few existing studies in the literature [38,48]. Moreover, all samples showed low values of lattice strain. SEM cross-sectional investigations of the CdTe nanostructured thin films allowed for the estimation of their corresponding thicknesses (583 nm for the CdTe sputtered at 70 W, 874 nm for the sample deposited at 80 W, 958 nm for the one at 90 W, and 983 nm for the one at 100 W) along with the general observation that all samples exhibited a compact aspect and good conformity, also in agreement with some previously reported studies [42,48]. The surface topography of the CdTe nanostructured thin films was analysed via the AFM technique and the typical surface morphology parameters RMS, Ssk, and Sku were determined in each case. As a general observation, all nanostructured thin films sputtered at each working power presented relatively low values of RMS, and there was a trend of linear increase in the RMS roughness parameter that was correlated with the increases in the RF sputtering power. Previous studies report much larger surface RMS values and larger variations in the RF plasma power [45,48]. The optical properties of the CdTe nanostructured thin films deposited on optical glass substrates were investigated via transmission and absorption spectroscopy. The transmission spectra of all RF-sputtered samples show that all the films possessed more than 65% transparency in the 800–1800 nm region; therefore, the material obtained in these specific conditions can be successfully employed as an absorber layer in different optoelectronic structures. Moreover, from these spectra, the optical thickness values of each CdTe thin film were also estimated (690 nm at 70 W, 941 nm at 80 W, 971 nm at 90 W, and 1078 nm at 100 W). A good agreement was observed with the ones determined by cross-section SEM analysis. Using the absorption spectra corresponding to each sample, the experimental band gap energies of the CdTe were computed by using the Tauc plot technique. The obtained values of the band gap energy (1.44 eV for the sample sputtered at 70 W, 1.47 eV for the one at 90 W, and 1.49 eV for the ones at 80 W and 100 W) are in line with the scientific literature. Comparing these results with the few existing studies regarding the effects of RF magnetron power on the properties of CdTe nanostructured thin films [38,42,45,48], in this study with higher RF power values than the other studies, the optical transmittance of the nanostructured thin films was higher for the respective thickness range, with a smaller variation in the thickness and estimated bandgap values that are less dependent on the RF magnetron power variation. This fact can be correlated with the larger crystallites in the films used in the present study, as well as their excellent thickness uniformity and surface morphology. The Ag/CdTe/Al “sandwich” structure was fabricated via VTE for Ag and Al layers in order to study the electrical properties of CdTe nanostructured thin films. The electrical investigation of the aforementioned structure consisted of recording the J–V characteristics in the dark. At forward bias, the dominant conduction mechanisms identified were the ohmic conduction at low injection levels (between 0.15 V and 0.5 V) and above the transition voltage (0.56 V), the SCLC, which, in the presence of exponential trap distribution at high injection levels (0.6–1.25 V), becomes dominant. At reverse bias, the thermionic emission of the majority charge carriers assisted by the field at the barrier (the Schottky mechanism) was identified as the transport mechanism. Accordingly, the thickness of the depletion layer (~10 nm) was evaluated and, by plotting the logarithmic dependencies for four temperatures, the Schottky barrier was found to be *Φ* ≈ 0.47 eV. Therefore, the Ag/CdTe/Al structure forms an ohmic contact at the Ag/CdTe interface, whereas at the Al/CdTe interface, there is a blocking contact. Considering the structural, morphological, optical, and electrical properties of the sputtered CdTe nanostructured thin films, and the impact that the varied RF power has upon them, one can conclude that, depending on the specific role CdTe plays when integrated into a structure, the deposition parameters, especially the deposition RF power, must be carefully chosen. The general conclusion is that the samples prepared at 90 W seem to have the best characteristics for use as an absorber layer due to their highest crystallinity, low lattice strain values, excellent bulk, and surface homogeneity, and 65% optical transmittance at 880 nm. However, all the presented CdTe films displayed characteristics that make them suitable to be integrated into optoelectronic devices, especially in solar cells for space applications.

## 5. Conclusions

The aim of the present work was to improve the quality of CdTe nanostructured thin films by using the RF magnetron sputtering (RF–MS) technique as a reliable deposition method, with the ability to control the deposition parameters more easily. This study focused on the investigation of the influence of RF power on the physical properties of thin CdTe films deposited on two types of substrates: optical glass and p-type Si. For this study, four different powers of 70, 80, 90, and 100 W have been selected. The results from investigations employing the techniques GIXRD, SEM, AFM, and UV–VIS spectroscopy, and determining the I–V characteristics, show the significant potential of these nanostructured thin films to serve as absorbent layers in ultra-thin-film solar cells. The results of this work add important experimental information to existing studies regarding the optimisation of the properties of CdTe material in the RF–magnetron sputtering growth process, which, up to now, has been proven as one of the most suitable techniques for achieving CdTe thin-film layers that can be used as absorber layers in real-life, large-scale applications. New relevant information released by the present study points towards 90 W RF power as the optimal power for obtaining a high crystallinity of ~1μm nanostructured thin films on p-Si and optical glass substrates with optical and electrical properties suitable for use as absorber layers. This study adds value to the existing international database on the specific subject.

## Figures and Tables

**Table 1:** Structural parameters of fabricated CdTe nanostructured thin films on p-type Si substrate by RF–magnetron sputtering before the thermal treatment.

Sample	D_ef_^(111)^ (nm)	${\langle \varepsilon^{2}\rangle }^{1/2}$	a (Å)
CdTe_70W	66	5.1 × 10^−3^	6.491
CdTe_80W	58	4.7 × 10^−3^	6.492
CdTe_90W	89	4.5 × 10^−3^	6.493
CdTe_100W	68	4.5 × 10^−3^	6.489

**Table 2:** Measured mean values of thickness for CdTe nanostructured thin films deposited on p-Si substrate estimated from cross-section SEM micrographs.

Sample	Measured Thickness (nm)
CdTe_70W	583 ± 74
CdTe_80W	874 ± 22
CdTe_90W	958 ± 34
CdTe_100W	983 ± 21

**Table 3:** Surface morphology parameters obtained for RF-magnetron-sputtered CdTe nanostructured thin films, where RMS represents the route mean square roughness, while Ssk and Sku denote skewness and excess kurtosis coefficients, respectively.

	Sample	CdTe_70W			CdTe_80W			CdTe_90W			CdTe_100W		
Area		RMS (nm)	Ssk	Sku	RMS (nm)	Ssk	Sku	RMS (nm)	Ssk	Sku	RMS (nm)	Ssk	Sku
1 × 1 μm^2^		0.622	0.145	−0.080	0.847	0.254	0.074	1.063	−0.144	−0.112	1.331	0.055	−0.399
5 × 5 μm^2^		0.581	0.300	0.155	0.708	0.231	0.114	0.876	0.093	0.285	1.007	0.163	0.190
10 × 10 μm^2^		0.521	0.171	0.109	0.598	0.137	0.056	0.664	0.009	0.223	0.739	−0.001	−0.007

**Table 4:** Optical band gap energies for CdTe/optical glass nanostructured thin films.

Sample	Thickness (nm)	E_g_ (eV)
CdTe_70W/optical glass	736 ± 15	1.44
CdTe_80W/optical glass	941 ± 25	1.49
CdTe_90W/optical glass	971 ± 18	1.47
CdTe_1000W/optical glass	1078 ± 28	1.49

## Data Availability

The raw and processed data required to reproduce these findings cannot be shared at this time due to technical or time limitations. The raw and processed data will be provided upon reasonable request until the technical problems have been solved.
